# Ruspini's Styptic

**Published:** 1831-04-01

**Authors:** 


### VIII. RUSPINI'S STYPTIC.

Dr. A. T. Thomson has announced the discovery of the composition of this nostrum, and we hope he may be found correct, for, unquestionably the medicine is one of the most powerful restrainers of hæmorrhage which we possess. Its price is enormous, and therefore its utility—indeed its use, is greatly curtailed. Without going into the process by wbich Dr. T. arrived at his conclusions, we may observe that he considers *Gallic Acid* as the active principle of this styptic. There are in it, he remarks, minute quantities of opium and sulphate of zinc, but they can have no operative effect. Dr. T. avers that the vehicle is alcohol, with a small quantity of rose-water to give it odour. This observation induces us to fear that there is some mistake, or that more than one kind of Ruspini's styptic is in the market. That which we examined a few months ago, while exhibiting it in a case of internal hæmorrhage, was insipid and tasteless as spring water. It had no appearance of alcohol. Dr. T. informs us that alcohol dissolves one fourth of its weight of gallic acid—and consequently that, "an excellent styptic may be, at any time, extemporaneously prescribed." "In cases of hæmaturia the addition of some gallic acid to a tincture of uva ursi, will be found to answer every indication which can be expected from the employment of astringents in this disease." Dr. T. thinks the gallic acid is taken into the circulation, and passes undecomposed through the kidneys, so as to reach the bleeding vessels wherever situated.—*Med. and Phys. Journ.*

